# SARS-CoV-2 Epitopes following Infection and Vaccination Overlap Known Neutralizing Antibody Sites

**DOI:** 10.34133/2022/9769803

**Published:** 2022-07-09

**Authors:** Li Yang, Te Liang, Lane M. Pierson, Hongye Wang, Jesse K. Fletcher, Shu Wang, Duran Bao, Lili Zhang, Zhen Huang, Wenshu Zheng, Xiaomei Zhang, Heewon Park, Yuwen Li, James E. Robinson, Amy K. Feehan, Christopher J. Lyon, Jing Cao, Lisa A. Morici, Chenzhong Li, Chad J. Roy, Xiaobo Yu, Tony Hu

**Affiliations:** ^1^Center for Cellular and Molecular Diagnostics, Tulane University School of Medicine, 1430 Tulane Ave., New Orleans, LA 70112, USA; ^2^Department of Biochemistry and Molecular Biology, Tulane University School of Medicine, 1430 Tulane Ave., New Orleans, LA 70112, USA; ^3^Beijing Key Laboratory for Forest Pest Control, Beijing Forestry University, Beijing 100083, China; ^4^State Key Laboratory of Proteomics, Beijing Proteome Research Center, National Center for Protein Sciences-Beijing (PHOENIX Center), Beijing Institute of Lifeomics, Beijing 102206, China; ^5^Hayward Genetics Center, Department of Pediatrics, Tulane University School of Medicine, 1430 Tulane Ave., New Orleans, LA 70112, USA; ^6^Department of Pediatrics, Tulane University School of Medicine, 1430 Tulane Ave., New Orleans, LA 70112, USA; ^7^Infectious Disease Department, Ochsner Clinic Foundation, New Orleans, LA 70121, USA; ^8^University of Texas Southwestern Medical Center, 5323 Harry Hines Blvd., Dallas, TX 75390, USA; ^9^Department of Microbiology & Immunology, Tulane University School of Medicine, 1430 Tulane Ave., New Orleans, LA 70112, USA; ^10^Division of Microbiology, Tulane National Primate Research Center, 18703 Three Rivers Road, Covington, LA 70433, USA

## Abstract

Identification of epitopes targeted following virus infection or vaccination can guide vaccine design and development of therapeutic interventions targeting functional sites, but can be laborious. Herein, we employed peptide microarrays to map linear peptide epitopes (LPEs) recognized following SARS-CoV-2 infection and vaccination. LPEs detected by nonhuman primate (NHP) and patient IgMs after SARS-CoV-2 infection extensively overlapped, localized to functionally important virus regions, and aligned with reported neutralizing antibody binding sites. Similar LPE overlap occurred after infection and vaccination, with LPE clusters specific to each stimulus, where strong and conserved LPEs mapping to sites known or likely to inhibit spike protein function. Vaccine-specific LPEs tended to map to sites known or likely to be affected by structural changes induced by the proline substitutions in the mRNA vaccine's S protein. Mapping LPEs to regions of known functional importance in this manner may accelerate vaccine evaluation and discovery of targets for site-specific therapeutic interventions.

## 1. Introduction

COVID-19 remains a global pandemic due to the failure of social containment efforts, lack of widespread vaccination, and emergence of more infectious variants. New vaccine and therapeutic approaches are needed to continue to combat the pandemic and mitigate its pathologic effects, and their development would benefit from new means to identify SARS-CoV-2 protein motifs that could function as drug targets.

Current drug development efforts have focused on screening candidate antibodies isolated from vaccinated individuals [1, 2] and animal models [3–6] and COVID-19 patients [7, 8] for their ability to serve as neutralizing antibodies. Subsequent low-throughput studies are then required to identify their binding sites, which may involve linearly dispersed amino acids that cluster in tertiary structures. Such nonlinear epitopes can complicate epitope definition and vaccine development approaches that target them. Several studies have thus employed immunoinformatic analysis to identify candidate LPEs for antibody screening [9–12] or attempted to identify LPE targets of COVID-19 patient antibodies [13–16], due to the relative simplicity of generating and screening monoclonal antibodies to LPEs versus epitopes derived from secondary or tertiary protein structure.

Since the trimeric SARS-CoV-2 spike protein is required for cell entry and is a major antibody target following SARS-CoV-2 infection [17, 18], most vaccines [1, 2] and monoclonal therapeutics [19, 20] target this protein or its specific epitopes. Several studies have focused on epitopes that map to the spike protein receptor binding domain (RBD), since antibodies that target this region have been shown to attenuate interaction with angiotensin converting enzyme-2 (ACE2) to inhibit cell entry [21–25]. However, antibodies to epitopes in the spike protein N-terminal domain (NTD) also elicit virus neutralization effects [13, 21, 26, 27], indicating that additional regions influence cell entry. Monoclonal antibody therapies that target one versus multiple epitopes also have reduced effectiveness [28, 29], indicating the need for more such targets, particularly since new mutations may attenuate the effectiveness of therapies to existing targets [30]. Further studies are also needed to determine: (1) the epitope overlap of immune responses of NHPs and patients following SARS-CoV-2 infection; and (2) whether antibody responses induced by SARS-CoV-2 infection and vaccination with the modified spike protein of the Pfizer-BioNTech and Moderna RNA vaccines are similar, particularly with regard to their neutralizing antibody responses.

We thus employed SARS-CoV-2 proteome microarrays to detect LPEs recognized by antibodies produced by SARS-CoV-2-infected NHPs, COVID-19 patients, and individuals vaccinated with the Pfizer-BioNTech and Moderna RNA vaccines. This analysis revealed substantial overlaps between LPEs detected in SARS-CoV-2-infected NHPs and patients, and vaccinated individuals, albeit with cohort-specific LPEs, several of which matched sites recognized by known neutralizing antibodies.

## 2. Results

### 2.1. Microarray Mapping of the NHP IgM Response to SARS-CoV-2 Infection

**Figure 1:**
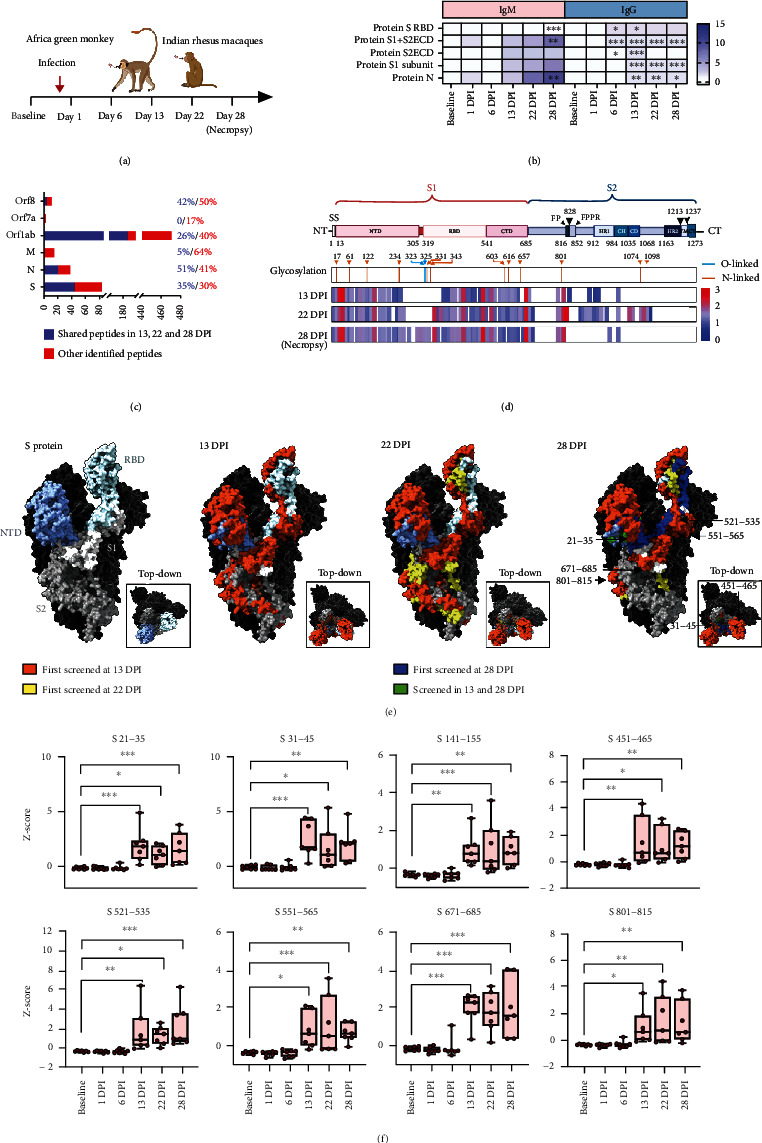
Proteomic microarray mapping of the antibody response to SARS-CoV-2 infection in nonhuman primates. (a) Schematic of experimental time points for the four AGMs and three RMs analyzed in this study. (b) Heatmap of the mean IgM and IgG signal for the indicated SARS-CoV-2 proteins and protein fragments. (c) Number and percentage of unique peptides detected by IgM binding from 13 to 28 dpi (blue) or at least one of these times (red). (d) Schematic and heatmap indicating the alignment of peptides detected at the indicated times with S protein features and their mean signal intensity minus signal intensity at baseline timepoints. O- and N-linked glycosylation positions are numbered and, respectively, marked by vertical blue and orange lines. (e) 3D structural maps of the SARS-CoV-2 S protein indicating the N-terminal domain (NTD), receptor binding domain (RBD), S1, and S2 peptide sequences and the corresponding peptide sequences bound by IgM at the indicated time points (PDB:6VYB). (f) Reproducibility of IgM signal trends for detected S protein peptides. Graphs indicate each individual value, minimum, lower quartile, median, upper quartile, and maximum, ^∗^*p* < 0.05, ^∗∗^*p* < 0.01, ^∗∗∗^*p* < 0.001, by repeated measure ANOVA with Dunnett multiple comparison test (*n* = 7) (created with biorender.com).

Plasma from NHPs subjected to SARS-CoV-2 aerosol exposure was hybridized to a microarray containing SARS-CoV-2 structural proteins and overlapping peptides to evaluate SARS-CoV-2-specific antibody changes over time (Figure 1(a) and Table S1). NHP plasma samples collected before infection and 1-day postinfection (dpi) revealed weak IgM and IgG signal for SARS-CoV-2 protein fragments (Figure 1(b)) that increased by 6 dpi with differential intensity and significance. Strong but variable IgM signal tended to increase to achieve significance at 28 dpi, while weaker but less variable IgG signal was significant by 6 dpi and tended peak and decline by 13 dpi.

IgM weakly detected the S protein RBD and S2 extracellular domain (ECD), but strongly recognized the S protein S1 subunit, S1 + S2 ECD region, and N protein. The strongest IgG binding was detected to the S protein RBD followed by slightly delayed and weaker signal for the S1 + S2 ECD (30~70% of RBD mean *z*-score) and N protein (45~70%), while reduced and transient signal was detected for the S1 subunit and S2 ECD regions (~20%). Similarly, IgM also detected more SARS-CoV-2 peptides, with stronger signal than IgG (Table S2). Since IgG antibodies derive from the IgM population by isotype switch recombination and since multimeric antibodies, and particularly IgM, play major roles in SARS-CoV-2 neutralization [31–33], all subsequent studies focused on IgM epitopes.

No IgM binding was detected to peptides derived from Orf3a, Orf6, or Orf10 proteins (Figure 1(c)), but IgM detected a subset of Orf7a peptides (17%), and more peptides from Orf1ab, M, and S (65–68%) and Orf8 and N (92–93%) proteins, with a subset of Orf1ab, Orf8, M, N, and S peptides (26–51%) revealing consistent signal at 13–28 dpi.

Rhesus macaque (RM) IgM recognized more peptides than IgM from African green monkeys (AGM) (Figure S1b), with most AGM IgM targets (5–48%) representing a subset of those detected by RM IgM, and unique AGM IgM antibodies representing only 2%, 6%, and 7% of the detected Orf1ab, S, and Orf3a/7a/8 protein epitopes.

S protein LPEs detected at successive dpi were mapped onto its structure to identify structural regions recognized during the IgM response, finding that these LPEs tended to cluster at adjacent sites in its RDB, S1 N-terminal and C-terminal domains, and S2 subunit (Figure 1(d)). Fourteen LPEs were detected at or near reported S protein N- or O-glycosylation sites, which were not glycosylated on array peptides, indicating that these modifications did not inhibit IgM recognition of this region.

Most S1 LPEs were consistently detected at similar intensity from 13 dpi onward, with a tendency to detect adjacent epitopes with additional time postinfection (Figure S1a and Figures 1(d) and 1(e)). S2 region LPEs were less consistent, with most found at 13 dpi and 22 dpi no longer detected at 28 dpi infection. Notably, several LPEs detected with strong and persistent IgM signal mapped within or near S protein functional regions (Figure 1(f)), including the RBD (LPEs S451-465 and S521-535), the S1/S2 cleavage site (S671-685), a furin cleavage site adjacent to the fusion peptide (FP; S801-815), and a S1 NTD site (S141-155) recognized by an antibody (4A8) with strong neutralization activity [27].

### 2.2. Patients IgM Responses to SARS-CoV-2 Infection

Serum from COVID-19 patients and individuals assessed months before the COVID-19 pandemic was also applied to a protein/peptide microarray to detect IgM and IgG binding to SARS-CoV-2 structural proteins and overlapping LPEs, including variants associated with distinct SARS-CoV-2 isolates, and proteins from other human respiratory viruses (Figure S2b and Table S1).

**Figure 2:**
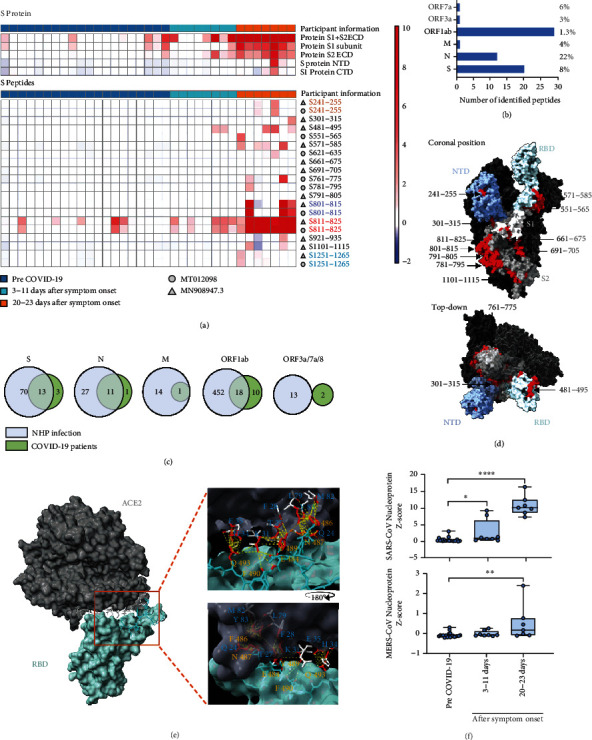
Proteomic microarray mapping of antibody responses in COVID-19 patients. (a) Heatmap of IgM signal *Z*-scores detected for SARS-CoV-2 proteins and peptides using serum collected pre-COVID-19 (blue, *n* = 20) or at 3-11 days (bright blue, *n* = 8) or at 20-23 days (orange, *n* = 7) after symptom onset in COVID-19 patients. Colored labels indicate signals detected from overlapping peptide sequences of SARS-CoV-2 strains MT012098 (circles) and MN908947.3 (triangles). (b) Number and percentage of peptides detected by IgM binding in COVID-19 patients. (c) Overlap of unique linear peptide epitopes detected with serum from SARS-CoV-2-infected NHPs and patients. (d) 3D structural map indicating IgM binding to linear peptide epitopes on the N-terminal domain (NTD), receptor-binding domain (RBD), S1, and S2 peptide sequence regions of the SARS-CoV-2 S protein (PDB:6VYB). (e) 3D structure indicating close interactions (yellow dash lines; ≤4.5 angstroms) between predicted contact residues (red) in a detected RBD linear peptide epitope (blue chain; S481-495) and ACE2 (white chain) (PDB:6LZG). (f) SARS-CoV and MERS-CoV N protein IgM signal for at the indicated times. Graphs indicate individual, minimum, lower quartile, median, upper quartile, and maximum values. ^∗^*p* < 0.05, ^∗∗^*p* < 0.01, ^∗∗∗^*p* < 0.001, ^∗∗∗∗^*p* < 0.0001, by nonparametric one-way ANOVA with Dunn's posttest.

Serum drawn from COVID-19 patients early (3-11 days) and late (20-23 days) after symptom onset revealed a markedly stronger IgM than IgG response (Figure S3a), similar to the NHP cohort. Surprisingly, some samples drawn before the COVID-19 pandemic revealed IgM binding to the S1+S2 ECD fragment, S1 subunit, and the S2 ECD (Figure 2(a)), likely reflecting IgM crossreactivity with similar epitopes on related *β* coronaviruses responsible for common human respiratory infections (e.g., OC43 and HKU1).

Strong IgM signals were detected for the S1+S2 ECD, S1, and S2 ECD subunits, while the S1 NTD and CTD regions had weak signal. IgG signal was also detected for the S1+S2 ECD and S1 subunit, but not other S protein regions. Similar to the NHP results, the human IgM response varied in its ability to consistently detect LPEs in different SARS-CoV-2 coding regions (Figure 2(b) and Table S3). No IgM binding was detected to Orf6, Orf8, or Orf10 peptides, but IgM detected a small percentage of the total Orf1ab, Orf3a, Orf7a, M, and S protein peptides (1.3%-8%) and larger fractions of the N protein (23%) peptides. LPEs detected in the COVID-19 patient cohort demonstrated variable overlap with NHP LPEs, which ranged from 92% to 81% to 64% for the N, S, and Orf1ab proteins, respectively. No overlap was observed among the Orf3a, Orf7a or Orf8 peptides, and a single M protein LPE was detected in both species (Figure 2(c)).

Multiple LPEs detected in the COVID-19 patient cohort matched peptides from more than one SARS-CoV-2 strain. Four of the twelve N protein sites detected had peptides from two strains, although only one of these duplicates yielded significant IgM signal (Table S4; N211-225). All 27 Orf1ab protein sites detected had variable overlaps with sequence from more than one strain, but significant IgM signal was detected by multiple peptides at only one of these sites, where three peptides shared an eleven residue consensus sequence overlap (Table S4; Orf1ab7041-7055). Similar to Orf1ab, all 16 S protein LPEs detected had variable sequence overlaps with more than one strain, with most aligned peptides being offset by, or varying at, a single reside. However, only four sets of these paired peptides were detected on the array (Table S4; S241-255, S801-815, S811-825, S1251-1265), and most differentially detected peptide sets (69%; 9 of 13) had charged or polar residues at their N- or C-terminus.

Most S protein LPEs detected (Figure 2(d)) localized to the S1 and S2 subunits with similar frequency (seven versus nine instances), with half (eight of sixteen) clustering within a 230 amino acid region between the S1 RBD and S2 FP motif. This differed from the NHP results, where most LPEs mapped to the S1 subunit (Figure 1(d)).

Similar to the NHP results, several LPEs detected in the COVID-19 cohort mapped at or near S protein regions with known functions, including its RBD, FP motif, and one of its two heptad repeats, including a RBD site (S481-495) detected in both the NHP and COVID-19 patient cohorts that is reported to directly interact with its ACE2 receptor [18, 34]. A structural model of the RBD:ACE2 interaction [34] indicated that six sidechains of this peptide (residues E484, F486, N487, Y489, F490, and F493) formed close associations (<4.5 angstroms) with nine ACE2 sidechains (Figure 2(e)). This S1 peptide sequence also partially overlapped the binding sites of two neutralizing antibodies (S2H13 [26] and F2B-2F6 [21]) isolated from COVID-19 patients, suggesting that this site is a common target of neutralizing antibodies. An ELISA analysis also indicated this peptide specifically bound ACE2 at low concentration versus a control peptide, and partially blocked ACE2 binding with the SARS-CoV-2 RBD at high concentration (Figure S4).

Strong IgM interactions with two LPEs near the RBD (S551-565 and S571-585) may also inhibit its interaction with ACE2 via steric hindrance, similar to an effect found with S14P5 LPE (S562-579) in another study [35]. A second IgM binding site at position S661-675 contained four residues (N657, N658, Y660, and E661) that contact furin protease to promote S protein cleavage and induce membrane fusion and cell entry [36]. Thus, antibody interaction at this site is likely to decrease virulence [36, 37]. Finally, two strong LPE sites (801-815 and 811-825) map near cleavage sites [38, 39] between S1/S2 and S2' upstream of the cell entry FP [39], and antibody recognition may therefore block cleavage or FP activity.

Most individuals, including those in the pre-COVID-19 pandemic group, revealed strong IgM signal for proteins from multiple common respiratory viruses, including multiple common human coronaviruses (HCoVs) and MERS-CoV (Figure S3b). Non-N protein MERS-CoV and SARS-CoV signals were not enhanced after SARS-CoV-2 infection, but IgM signal for several HCoV S proteins decreased at 20-23 days after symptom onset (Figure S3b).

### 2.3. Microarray Mapping of IgM Responses to SARS-CoV-2 Vaccination

SARS-CoV-2 infection and vaccination may produce IgM repertoires that differ in coverage or relative affinity for specific S protein sites, since the mRNA vaccines encode a modified S protein locked into a prefusion conformation to enhance production of antibodies that can neutralize its fusion activity [1, 2, 40]. We therefore analyzed the antibody response of a small longitudinal cohort of vaccinated individuals before vaccination and at defined intervals after receipt of the first and second vaccine dose.

**Figure 3:**
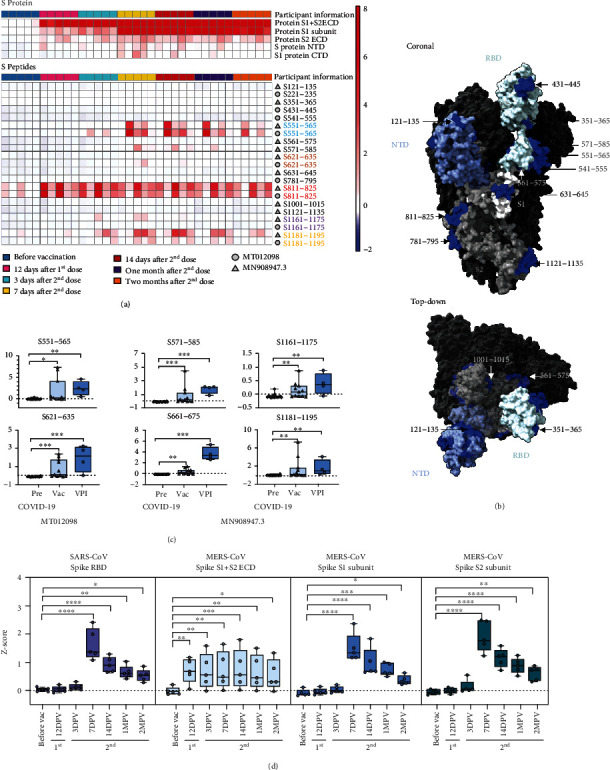
Proteomic microarray mapping of antibody responses in SARS-CoV-2 RNA vaccine participants. (a) Heatmap of IgM signal *Z*-scores for the SARS-CoV-2 proteins and peptides using serum collected at the indicated times before and after vaccination (*n* = 5). Colored labels indicate signals detected from overlapping peptide sequences of SARS-CoV-2 strains MT012098 (circles) and MN908947.3 (triangles). (b) 3D structural map (PDB:6VSB) indicating IgM binding to linear peptide epitopes in the N-terminal domain (NTD), receptor binding domain (RBD), S1, and S2 peptide sequence regions of the modified SARS-CoV-2 S protein (K986P and V987P) of the Pfizer and Moderna vaccines. (c) Relative IgM signal *Z*-scores for the strong linear peptide epitope signals in the vaccinated (Vac) (*n* = 11) and vaccinated postinfection (VPI) (*n* = 4) groups. (d) Relative IgM signal *Z*-scores for SARS-CoV and MERS-CoV S protein regions. Graphs indicate individual, minimum, lower quartile, median, upper quartile, and maximum values; ^∗^*p* < 0.05, ^∗∗^*p* < 0.01, ^∗∗∗^*p* < 0.001, ^∗∗∗∗^*p* < 0.0001, by nonparametric one-way ANOVA with Dunn's posttest (*n* = 5).

Strong IgM responses to S1 + S2 ECD and S1 proteins were, respectively, detected by 12 days after the first vaccine dose and 3 days after the second dose (Figure 3(a)), with weaker responses to S2 ECD and S1 NTD and CTD proteins detected by 12 and 7 days after the first and second dose. Similar to the COVID-19 cohort results, IgG binding was detectable only for the S1+S2 ECD and S1 proteins, peaking by 7-14 days after the second dose and rapidly declining thereafter (Figure S5).

S protein LPE sites (Figure 3(b) and Table S5) revealed a pattern substantially different form the COVID-19 cohort, sharing only five unique peptides among these groups (S551-565, S571-585, S621-635, S781-795, and S811-825). Peptides detected in these groups tended to form near-continuous linear clusters (Table S6), with five peptides in the COVID-19 cohort forming two clusters (S661-705 and S761-825) and nine peptides in the vaccine cohort forming three clusters (S541-585, S621-645, and S1121-1195).

The COVID-19 cohort LPE clusters mapped to the S1-S2 junction and overlapped the S2 region containing the FP motif, while the vaccine cohort LPE clusters mapped near the RDB and within the S1 CTD and S2 heptad repeat 2 (HR2). However, peptides in three of these five clusters were detected in both cohorts, implying preferential rather than absolute differences in site recognition, supported by the NHP cohort results, which detected multiple peptides in all but one of these clusters.

IgM LPEs detected in a second, crosssectional vaccine cohort of individuals who had serum collected 7-30 days after their second vaccine dose, including four individuals vaccinated post-SARS-CoV-2-infection (VPI), detected all the peptides found in the first vaccine cohort, and one peptide (S661-675) originally detected only in the COVID-19 cohort. VPI individuals also had more peptides with stronger signals, including five of the six peptides detected in both the COVID-19 and vaccine cohorts (Figure 3(c) and Table S5).

Similar to the COVID-19 cohort, most vaccine cohort subjects revealed strong IgM signal for proteins of common respiratory viruses, including several common HCoVs, with similar responses detected pre- and postvaccination (Figure S5c). Vaccination produced transient increases in IgM binding to MERS-CoV and SARS-CoV S proteins, peaking at 7 days after the second vaccine dose, although a MERS-CoV S1+S2 ECD response not altered by vaccination likely reflect nonspecific recognition of this ECD region (Figure 3(d)).

### 2.4. Epitope Overlap in Vaccinated and SARS-CoV-2-Infected Individuals

**Figure 4:**
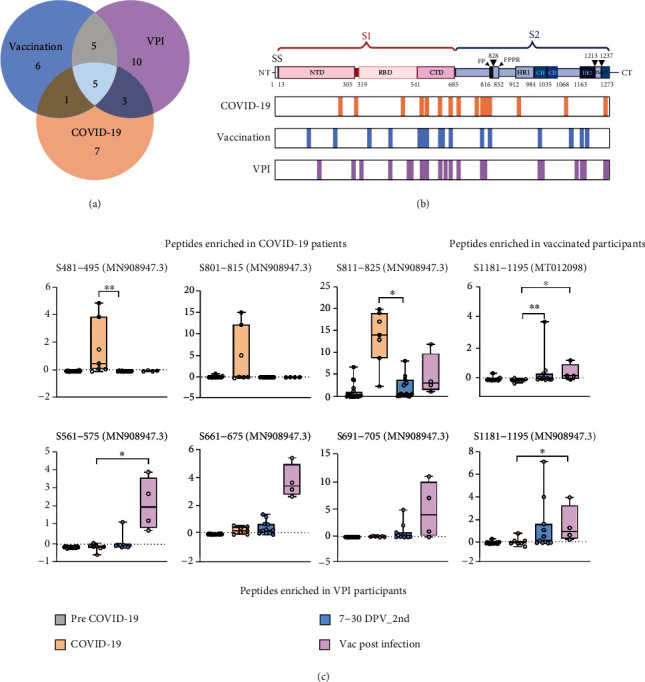
Difference in IgM linear peptide epitopes detected in the COVID-19, vaccinated, and VPI groups. (a) Venn diagram of the overlap between IgM linear peptide epitopes detected in SARS-CoV-2 patients (COVID-19), vaccinated individuals (Vac), and individuals vaccinated postinfection (VPI). (b) SARS-CoV-2 S protein schematic indicating sites of IgM linear peptide epitopes specific for and shared among the indicated groups. (c) Relative *Z*-score differences for IgM linear peptide epitopes preferentially detected in COVID-19 (*n* = 7), vaccinated (*n* = 11), and VPI (*n* = 4) individuals. Graphs indicate each individual, minimum, lower quartile, median, upper quartile, and maximum values; ^∗^*p* < 0.05, ^∗∗^*p* < 0.01, ^∗∗∗^*p* < 0.001, ^∗∗∗∗^*p* < 0.0001, by nonparametric one-way ANOVA with Dunn's posttest (created with biorender.com).

Substantial LPE overlap was detected among the COVID-19, vaccine, and VPI groups, although this varied by groups with only five LPEs shared among all three (Figure 4(a) and Table S7). Each group revealed overlapping coverage, with LPEs detected in a single group tended to cluster in the S1 NTD and RBD, and the S2 HR1 to HR2 regions (Figure 4(b) and Table S7). LPEs detected in both the COVID-19 and VPI groups clustered in the S2 region outside defined functional domains, while those found in both the vaccinated and VPI groups localized to multiple functional sites, including the RBD, the HR1, and the central helix (CH) domains, as well as two S1 CTD sites. Finally, of the five LPEs detected in all three groups, all but one mapped to sites within the S1 subunit CTD, with the remaining LPE mapping to a site within the S2 NTD.

Seven of the 12 top ranked LPEs, as defined by *Z*-score and *p* value, were similarly detected in all three groups, while three were exclusively in the COVID-19 group and two were preferentially detected in both vaccine groups (Figure 4(c)). The three peptides detected in the VPI group but not the COVID-19 group failed to significantly differ from the vaccine group due to signal variability and sample size. These three LPEs mapped to the RBD and adjacent to the FP, while the two expressed in the two vaccinated groups mapped near the S1/S2 junction or HR2. Thus, LPEs detected in SARS-CoV-2-infected and vaccinated individuals map to regions of functional importance, with differences that likely reflect a conformational change in the mRNA vaccine-encoded S protein.

## 3. Discussion

More comprehensive detection and characterization of epitopes that can neutralize critical SARS-CoV-2 functions is necessary to guide the design of new vaccines and monoclonal antibody therapeutics, particularly in the context of emerging variants. However, current approaches are limited in their ability to precisely define epitopes or exhibit screening bias. Microarray screening permits rapid characterization of antibody responses to SARS-CoV-2 variants and vaccines, but the overlap between NHP and human responses, including epitope coverage and site preference, remains unclear. We found that most (13 of 16) S protein IgM LPEs detected in the COVID-19 cohort matched LPEs detected in the NHP cohort. LPEs strongly detected in both species tended to localize to functionally important S protein regions and align with reported neutralizing antibody binding sites. Vaccine-associated IgM epitopes revealed a slightly altered distribution pattern, although at least one strongly detected LPE in each cluster was shared among these groups, suggesting that these differences may not affect IgM recognition and inhibition of functional sites that map near to or overlap these LPE clusters.

This study has limitations. First, while peptide microarray studies can identify LPEs with complete sequence coverage, precise localization, and low selection bias, they cannot detect epitopes without extended linear sequence, although these may account for <25% of those induced by recombinant antigens [41]. Sequence fragmentation among overlapping peptides could also miss antibodies, but this effect may be small since 85% of antibodies that bind LPEs recognize ≥5 contiguous amino acids [42], and most recognize epitopes defined by seven to nine amino acids [43].

Second, it has constrained statistical power, reducing its ability to detect LPEs recognized by lower abundance or lower affinity antibodies, particularly in the COVID-19 cohort since unknown exposure doses, infection intervals, and virus strains all add variability. These issues are difficult to address due to the difficulty of performing large NHP studies, recruiting large, well-characterized vaccine-naïve COVID-19 patient cohorts with virus sequence information, and recruiting and retaining significant numbers of vaccine-naïve individuals for serial evaluation. Finally, this analysis focused on IgM LPEs given their greater abundance and signal intensity than IgG LPEs, potentially due to the greater avidity of IgM during competition for the same sites. Immune selection may favor IgGs that recognize nonlinear SAR-CoV-2 epitopes or LPEs constrained by secondary structure. Microarray peptides lack this structural stability, and this should negatively affect antibody-peptide association and dissociation rates and therefore preferentially destabilize IgG versus IgM binding due to its inherently lower avidity. Sample volume limitations did not permit a separate IgG analysis using serum IgM-depleted serum, and an IgG analysis would therefore require more sample from new cohorts. However, while most studies have focused on IgG epitopes, the IgG population present prior to affinity maturation is derived from the IgM repertoire and multimeric antibodies, and particularly IgM, play major roles in SARS-CoV-2 neutralization [31–33].

Conserved LPE sites detected in our study tended to map to three major S protein regions: its RBD, cleavage sites, or cell-entry-associated sequences (e.g., FP, HR1, and HR2 regions). NHP cohort LPEs revealed near complete RBD coverage, and multiple antibodies biding this region can block viral entry by inhibiting RBD interactions with ACE2 [18, 35, 44, 45]. One LPE (S481-495) found in the NHP and COVID-19 cohorts partially overlapped the binding site of two neutralizing antibodies isolated from COVID-19 patients, S2H13 [26] and F2B-2F6 [21].

Several LPEs also mapped to cleavage sites associated with cell entry [37, 46], including polybasic and multibasic furin cleavage sites partially cleaved during viral production to promote membrane fusion and viral transmission [17, 37, 47, 48]. This included a peptide (S661-675) that mapped to a furin site between the S1 and S2 subunits, where antibody binding can block subunit separation and inhibit conformational changes associated with membrane fusion [36, 37]. Another LPE (S801-815) mapped to an FP-adjacent site, where antibody binding could inhibit cleavage required for cell entry, while another LPE containing the FP sequence had strong signal following both infection and vaccination. Stronger signal detected in the former group for both LPEs could reflect a conformational effect of the modified mRNA vaccine S protein, since this region normally packs tightly around an internal disulfide bond, but adopts a looser conformation in the vaccine since the K986P mutation alters the net charge at the trimer interface [39]. Several detected peptides also mapped to HR1 and HR2, which interact to promote the colocalization and fusion of the viral and cell membranes [38, 49]. Steric or conformational antibody binding effects, however, should attenuate virus infectivity, similar to the peptide EK1 fusion inhibitor, which interacts with HR1 to block HR2 binding, membrane fusion, and cell entry [50, 51].

Monoclonal antibody therapy effectiveness remains under evaluation despite significant in vitro data supporting the ability of such antibodies to reduce SARS-CoV-2 infectivity. SARS-CoV-2 variants could compromise their efficacy, however, and multiple neutralizing antibodies targeting distinct epitopes may be required to circumvent escape mutations [29, 52, 53]. Since the RBD exhibits substantial mutation, epitopes that map to other effector regions may be proved useful as alternate targets for neutralizing antibodies or inhibitors. This could include LPEs conserved among infected and vaccinated individuals that map to CTD1 (S551-565; S561-575), and to CTD2 (S621-635; S661-675). These regions are located below the RBD and against the S2 and NTD regions in secondary structure [54], and thus antibody binding to either site could alter structural interactions that affect S protein activity. In support of this hypothesis, CTD1 appears to mediate an interaction between the RBD and FPPR regions to regulate structural rearrangements that favor membrane fusion activity [39], while a CTD2 interaction may stabilize cleaved S1-S2 protein complexes to prevent S1 dissociation [55] and thus subsequent S2 structural rearrangements involved in membrane fusion [56, 57].

Our findings indicate that microarray screening can provide valuable information about the adaptive immune response to SARS-CoV-2 infection and vaccination by permitting high-resolution mapping of LPEs. Specifically, our results indicate a strong correspondence among LPEs detected in NHPs and patients following SARS-CoV-2 infection and that strongly detected LPEs tend to be conserved and map to functionally important regions of the S protein, with those revealing differential intensity among the vaccinated and infected groups aligning with potential differences in protein conformation among the native and recombinant protein. Taken together, we believe these results indicate the strong potential of employing microarray mapping data with structural and function information to identify candidate targets for therapeutic interventions.

## 4. Materials and Methods

### 4.1. Sample Collection

#### 4.1.1. SARS-CoV-2-Infected Nonhuman Primates

Plasma samples obtained from a cohort of SARS-CoV-2-infected NHPs (Table S8) were analyzed in this study. This cohort was generated at the Tulane National Primate Research Center using an established model of SARS-CoV-2 infection. Briefly, a total of seven male NHPs aged 7 to 11 years were subjected to aerosol inoculation with the SARS-CoV-2 isolate USA-WA1/2020 (CDC). Four African Green Monkeys (AGMs) were exposed to a dose of 1 × 10^4^ TCID50, and three Indian Rhesus Macaques (IRMs) were exposed to a dose of 0.5 × 10^4^ TCID50. The animals were evaluated by twice daily monitoring for 28 days after infection by veterinary staff, and blood samples were collected from all animals 7 days prior to SARS-CoV-2 exposure and at 1, 6-, 13-, 22-, and 28 days postinfection.

#### 4.1.2. COVID-19 Patient Cohort

Blood samples were collected from thirty-five patients seen at Weill Cornell Medicine (Table S9). This cohort included fifteen individuals who were hospitalized with COVID-19 and twenty who were seen before 2019, whose blood samples were designated pre-COVID-19 samples. Of the fifteen COVID-19 patients in this cohort (4 women and 11 men; aged 35 to 87 years), eight had blood collected 3-11 days after symptom onset, and the remaining seven each had blood collected at 20-23 days after symptom onset.

#### 4.1.3. SARS-CoV-2 mRNA Vaccine Cohort

Five participants scheduled for vaccination with the Pfizer-BioNTech COVID-19 mRNA vaccine were enrolled in a longitudinal study to evaluate the dynamic response of linear epitopes produced during the subsequent immune response (Table S9). Fingertip blood samples were collected before vaccination, at 12 days after the first vaccine dose, and at 3, 7, and 14 days, and 1 and 2 months after the second dose. Fifteen individuals vaccinate with either the Pfizer-BioNTech or Moderna COVID-19 mRNA vaccine were enrolled in a second study to independently confirm the reproducibility of major linear epitopes produced following vaccination (Table S9). Lithium heparin plasma specimens were stored at 4°C after collection for up to 72 hours and were then stored at -80°C before analyzing. Fifteen study participants had blood samples collected between 7 and 30 days after receipt of their second vaccine dose, and four of these individuals had documented COVID-19 cases prior to their first vaccine dose.

### 4.2. Microarray Design

Two SARS-CoV-2 proteome microarray libraries were employed in this study: a one-strain SARS-CoV-2 microarray library and an international SARS-CoV-2 proteome microarray library that contained peptide sequences from several SARS-CoV-2 strains.

The one-strain SARS-CoV-2 proteome microarray library contained S, N, and E proteins and 966 peptides covering the entire SARS-CoV-2 genome (MN908947 strain), each containing 15 amino acids with a 5 amino acid overlap among each sequential peptide (Table S1 and Table S10).

The international SARS-CoV-2 proteome microarray library contained S, N, and E proteins and/or fragments of these proteins and 966 peptides covering the entire SARS-CoV-2 genome (MN908947.3 strain), each containing 15 amino acids with a 5 amino acid overlap among each sequential peptide. This array also included 1697 peptides from 52 other SARS-CoV-2 isolates collected at multiple international locations, most of which had demonstrated sequence offsets or amino acid variations with the MN908947.3 strain. This array also contained proteins and/or fragments encoded by several other respiratory viruses which might demonstrate crossreactivity with SARS-COV-2 specific antibodies (e.g., human respiratory syncytial virus, influenza A/B, dromedary camel coronavirus, human-CoV, MERS-CoV, and SARS-CoV) (Table S1, Table S10, and Figure S2b).

### 4.3. SARS-CoV-2 Antibody Screening on the Proteome Microarray Platforms

All microarray hybridization steps were performed in a humidified chamber to reduce potential artifacts arising from unequal sample evaporation. Microarray chips were blocked at RT for 10 min with 400 *μ*L 5% filtered skim milk, incubated for at 20 min at RT with gentle rocking with 4 *μ*L NHP plasma diluted into 400 *μ*L 5% skim milk, and then, washed three times with 1× PBS supplemented with 0.05% Tween-20 (0.05% PBST) for 5 min at each wash. Chips were then incubated for 30 min at RT with 4 *μ*g/mL fluorescent-labeled monkey-specific IgG and IgM secondary antibodies [DyLight 650-conjugated Goat Anti-Monkey IgG (H + L) and DyLight 550-conjugated IgM] (Novus Biologicals), diluted in 400 *μ*L 5% skim milk, and washed three times with 0.05% PBST for 5 min at each wash and two times with deionized water for 1 min at each wash. Proteome microarray chips were then scanned using an Agilent microarray scanner, and the fluorescence signal intensity of each spot was extracted using GenePix Pro7 software (Molecular Devices). Reproducibility of antibody detection for the one-strain SARS-CoV-2 proteome microarray was determined by comparing the two spots for one peptide in one array and same peptide spot from two arrays incubated with the same sample (Figure S2a and Table S10). It is the same process for the screening of SARS-CoV-2 antibodies from infected/vaccinated individuals on the international SARS-CoV-2 proteome microarray with fluorescent-labeled human-specific IgG and IgM secondary antibodies [Cy3-conjugated AffiniPure Donkey Anti-Human IgG (H+L) and Alexa Fluor647-conjugated AffiniPure Goat Anti-Human IgM (Fc5*μ* fragment specific)] (Jackson ImmunoResearch) (Incubation volume: 3 mL) (Figure S2b). Reproducibility of antibody detection for the international SARS-CoV-2 proteome microarray was determined by comparing the two spots for one peptide in one array and same peptide spot from two arrays incubated with the same sample (Figure S2c).

### 4.4. Immunofluorescence Assay

In the immunofluorescence assays performed to evaluate potential crossreactivity of the anti-human IgG (H+L) and IgM antibodies used in these analyses, serial dilutions (2, 0.5, and 0.125 *μ*g/mL) of unlabeled versions of these two antibodies were separately added to wells of 96-well high-binding plates and incubated at RT for 3 hours to allow complete binding. These plates were then blocked at RT for 1.5 hour with 2% (*w*/*v*) bovine serum albumin (BSA) in 0.05% PBST and washed three times with 0.05% PBST buffer, after which blocked wells were incubated for 1 hour at RT with 4 *μ*g/mL of the Cy3-conjugated AffiniPure Donkey Anti-Human IgG (H + L) and/or AlexaFluor 647-conjugated AffiniPure Goat Anti-Human IgM (Jackson ImmunoResearch) in 1% BSA PBST buffer. Wells were then washed three times with 0.05% PBST, after which their fluorescence intensity (Figure S2d) and images were captured using a Cytation 5 Imaging Multimode Reader (BioTek).

### 4.5. Spike Peptide Interaction with ACE2

Enzyme-linked immunosorbent assay (ELISA) plates used to evaluate the interaction of the S481-495 peptide and with recombinant human ACE2 were generated by incubating 96 well plates with 100 *μ*L of 4 *μ*g/mL goat anti-human IgG Fc (Jackson ImmunoResearch) overnight at 4°C overnight. These ELISA plates were then incubated for 2 hours with 100 *μ*L of 4 *μ*g/mL ACE2-Fc (GenScript) at 37°C and blocked by a 2-hour incubation at 37°C with 5% BSA in PBS. ELISA plate wells were then incubated with 100 *μ*L of PBS (zero control), two-fold serial dilutions (2000-3.90 ng/mL) of biotin-labeled S481 peptide, and 500 ng/mL of an N protein N161-175 negative control peptide (GenScript) in 1% BSA/0.05% PBST buffer for 1 hour at room temperature and washed with PBST. All wells but the zero control were then supplemented with 100 *μ*L of horseradish peroxidase- (HRP-) conjugated streptavidin (Thermo Scientific, 1 : 10000 dilution) in 1% BSA/0.05% PBST and incubated for 1 hour at 37°C, after which wells were then washed with PBST, incubated with 100 *μ*L 3,3′,5,5′ tetramethylbenzidine (TMB, Thermo Scientific) for 3~10 minutes, and supplemented with 100 *μ*L 2 N H_2_SO_4_ to stop the reaction, and absorbance at 450 nm was measured using a plate reader.

### 4.6. Spike Peptide Inhibition of the SARS-CoV-2 RBD and ACE2 Interaction

ELISA plates described above were incubated with 100 mL/well of 1% BSA/0.05% PBST buffer containing HRP-RBD (1 : 500) supplemented with or without 500 ng/mL of an N protein N161-175 negative control peptide (GenScript), S481-495 peptide (0-1000 *μ*g/mL), or 20 *μ*g/mL of unlabeled RBD protein for 1 hour at room temperature. All wells were then washed with PBST and incubated with TMB and analyzed as described above.

### 4.7. Structure Analysis

Epitope mapping and contact distance evaluation was performed using Chimera X1.2.5 software [58]. Three different 3D models of the SARS-CoV-2 spike protein were used to map epitopes. The first model (PDB:6VYB), which had one receptor-binding domain (RBD) in the “up” position, was used to map the relative position of epitopes detected in the NHP cohort and the COVID-19 patient cohort. The second model (PDB:6LZG) employed the spike protein RBD and the ACE2 receptor in their bound state to model an interaction between the RBD linear peptide epitope S481-495 and its ACE2 interaction site [34]. The third model (PDB:6VSB) was chosen to represent the structure of the SARS-CoV-2 spike protein used in the Pfizer and Moderna vaccines, where to proline substitutions (K986P and V987P) stabilize the spike protein with its RBD in the up position [40].

Using the model of the SARS-CoV-2 spike protein RBD bound to ACE2, van der Waals interactions between amino acid residues of these proteins were evaluated using the “Contacts” function in Chimera X1.2.5 Settings chosen for this analysis identified pairs of atoms with center − to − center distances ≤ 4.5 angstroms, ignored interactions between atoms four or fewer bonds apart, and included intermodel and intramodel interactions. Results from this analysis matched other published data on the interactions between the RBD and ACE2 [18, 34].

### 4.8. Statistics

Statistical methods employed for data analysis are defined in the corresponding figure legends, with additional detail provided below.

Microarray fluorescence signal intensity was standardized using the *Z*-score method. Raw fluorescence signal intensity for each peptide was determined as the mean signal intensity of each spot and then averaged across duplicate spots of each peptide. The data generated for all samples of an NHP was normalized together. For COVID-19 patients and vaccinated participants, however, data was standardized independently for any longitudinal samples.

Identification of peptides was detected by IgM and IgG binding. For the NHP samples, peptides detected by IgM and IgG were defined as those with *p* values < 0.05 from repeated measure ANOVAs with Dunnett's post hoc tests when at least four of the seven NHPs had values greater than the baseline + three times its standard deviation. For longitudinal COVID-19 vaccine samples, peptides detected by IgM and IgG were defined as those with *p* values < 0.05 from repeated measure ANOVAs with Dunnett's post hoc tests and mean values greater than the prevaccination sample mean + three times its standard deviation. For COVID-19 patients and vaccinated individuals without longitudinal samples, peptides detected by IgM and IgG were defined as those with *p* valves < 0.05 from parametric one-way ANOVA with Dunnett's post hoc test and mean values greater than the mean of the pre-COVID-19 group + three times its standard deviation. ANOVA tests were performed using the “multcomp” R software library and its “mvtnorm,” “survival,” “TH.data,” and “MASS” packages.

Pearson correlation coefficients analyzed using GraphPad Prism 8 software were used to compare the fluorescence intensity between duplicate spots within an array and the fluorescence intensity of corresponding spots between arrays when these arrays were incubated with the same samples. Nonparametric one-way ANOVAs with Dunn's posttests performed using GraphPad Prism were used to evaluate the difference between the vaccinated, vaccinated postinfection, and COVID-19 patient groups. Heatmaps indicating antibody responses to SARS-CoV-2 proteins and peptides were constructed using the “pheatmap” package in the R software suite. Graphs were generated with GraphPad Prism, and schematic diagrams were generated using BioRender.

### 4.9. Study Approval

The Institutional Animal Care and Use Committee of Tulane University reviewed and approved all the procedures for NHP experiments. The Tulane National Primate Research Center is fully accredited by the Association for Assessment and Accreditation of Laboratory Animal Care (AAALAC). All animals are cared for per the NIH Guide to Laboratory Animal Care. The Tulane Institutional Biosafety Committee approved the procedures for sample handling, inactivation, and removal from BSL3 containment.

All human subjects provided written informed consent before study participation approved by the institutional review board of Tulane University, Weill Cornell Medicine and the University of Texas Southwestern Medical Center.

## Data Availability

The microarray data used to support the findings of this study are available from the corresponding author upon request.

## Supplementary Materials

Supplementary Materials Figures S1: the IgM signal intensity of spike peptide positions with individual monkeys and relative abundance and overlap among IgM linear epitopes detected in the SARS-CoV-2 NHP models with different species, related to Figure 1. Figures S2: composition and performance of the one one-strain SARS-CoV-2 proteome microarray and the international SARS-CoV-2 microarray, related to Figures 1 and 2. Figures S3: heatmap of the mean IgM and/or IgG antibody signal detected for the indicated proteins and protein fragments of SARS-CoV-2 or other respiratory viruses using serum from the COVID-19 patient cohort, related to Figure 2. Figure S4: an S protein LPE consistently detected by antibodies present in SARS-CoV-2-infected NHPs and patients (S481-495) binds to ACE2 and blocks its interaction with the S protein RBD. Figures S5: heatmap of the mean IgM and/or IgG antibody signal detected for the indicated proteins and protein fragments of SARS-CoV-2 or other respiratory viruses using serum from five individuals in vaccine cohort with longitudinal samples, related to Figures 3(a) and 3(b). Table S6: S protein epitope clusters detected in NHP, COVID-19, and vaccinated participants. Table S7: IgM S peptide among the COVID-19 patients, vaccinated and VPI participants. Other supplementary materials for this manuscript include the following: dataset S1. Table S1: composition of the one-strain SARS-CoV-2 proteome microarray and the international SARS-CoV-2 proteome microarray, related to Figures 1–3. Dataset S2. Table S2: identified peptides specific to anti-IgM and anti-IgG from nonhuman primates infected with SARS-CoV-2, related to Figure 1. Dataset S3. Table S3: proteins and peptides detected by COVID-19 patient IgM and IgG, related to Figure 3. Dataset S4. Table S4: IgM binding to overlapping SARS-CoV-2 peptides in COVID-19 patients, related to Figure 2. Dataset S5. Table S5: IgM- and IgG-specific targets recognized by longitudinal vaccine participants and crosssection vaccinated participants. Dataset S6. Table S8: information for animals in the nonhuman primate SARS-CoV-2 infection cohort, related to methods and Figure 1. Dataset S7. Table S9: information for individuals in the COVID-19 cohort and vaccinated participants refers to method and Figures 2 and 3. Dataset S8. Table S10: source of reagents in this manuscript.
