# Supplementary material for: SARS-CoV-2 Epitopes following Infection and Vaccination Overlap Known Neutralizing Antibody Sites
Source: Research (Wash D C). 2022 Jul 9;2022:9769803. doi: 10.34133/2022/9769803 (PMC9297724; doi:10.34133/2022/9769803)
Supplement: Supplementary Materials — Figures S1: the IgM signal intensity of spike peptide positions with individual monkeys and relative abundance and overlap among IgM linear epitopes detected in the SARS-CoV-2 NHP models with different species, related to Figure 1. Figures S2: composition and performance of the one one-strain SARS-CoV-2 proteome microarray and the international SARS-CoV-2 microarray, related to Figures 1 and 2. Figures S3: heatmap of the mean IgM and/or IgG antibody signal detected for the indicated proteins and protein fragments of SARS-CoV-2 or other respiratory viruses using serum from the COVID-19 patient cohort, related to Figure 2. Figure S4: an S protein LPE consistently detected by antibodies present in SARS-CoV-2-infected NHPs and patients (S481-495) binds to ACE2 and blocks its interaction with the S protein RBD. Figures S5: heatmap of the mean IgM and/or IgG antibody signal detected for the indicated proteins and protein fragments of SARS-CoV-2 or other respiratory viruses using serum from five individuals in vaccine cohort with longitudinal samples, related to Figures 3(a) and 3(b). Table S6: S protein epitope clusters detected in NHP, COVID-19, and vaccinated participants. Table S7: IgM S peptide among the COVID-19 patients, vaccinated and VPI participants. Other supplementary materials for this manuscript include the following: dataset S1. Table S1: composition of the one-strain SARS-CoV-2 proteome microarray and the international SARS-CoV-2 proteome microarray, related to Figures 1–3. Dataset S2. Table S2: identified peptides specific to anti-IgM and anti-IgG from nonhuman primates infected with SARS-CoV-2, related to Figure 1. Dataset S3. Table S3: proteins and peptides detected by COVID-19 patient IgM and IgG, related to Figure 3. Dataset S4. Table S4: IgM binding to overlapping SARS-CoV-2 peptides in COVID-19 patients, related to Figure 2. Dataset S5. Table S5: IgM- and IgG-specific targets recognized by longitudinal vaccine participants and crosssection vaccinated [file 9769803.f1.zip › Supplementary_Figures_Tables.docx]

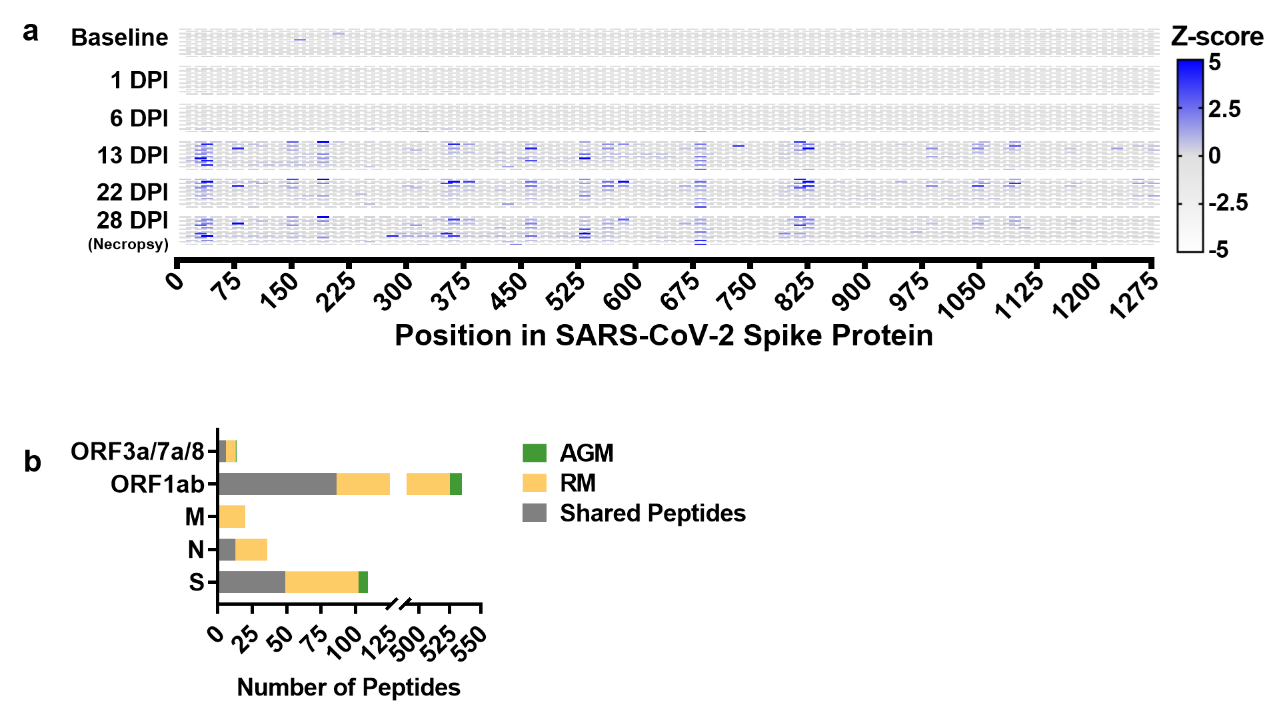


**Figure S1. The IgM signal intensity of spike peptide positions with individual monkeys and relative abundance and overlap among IgM linear epitopes detected in the SARS-CoV-2 NHP models with different species, related to Fig.1.**

**a**. Normalized data of the signal intensity for spike peptide positions, screened with anti-IgM, within SARS13

CoV-2 spike protein sampled from 7 individual monkeys at multiple time points before and post-infection. Each monkey’s data is represented as two horizontal lines, each line showing signals for peptides that comprise offset multiples of 15 amino acids (ie, 1-15, 21-35, 41-55 vs 11-25, 31-45, 51-65). (n = 7)

**b.** Relative abundance and overlap among IgM linear epitopes detected in the SARS-CoV-2 NHP models. Bar graphs indicates the number of peptides uniquely detected in serum from SARS-CoV-2-infected RM (yellow) and AGM (green) or shared between the two species (gray).


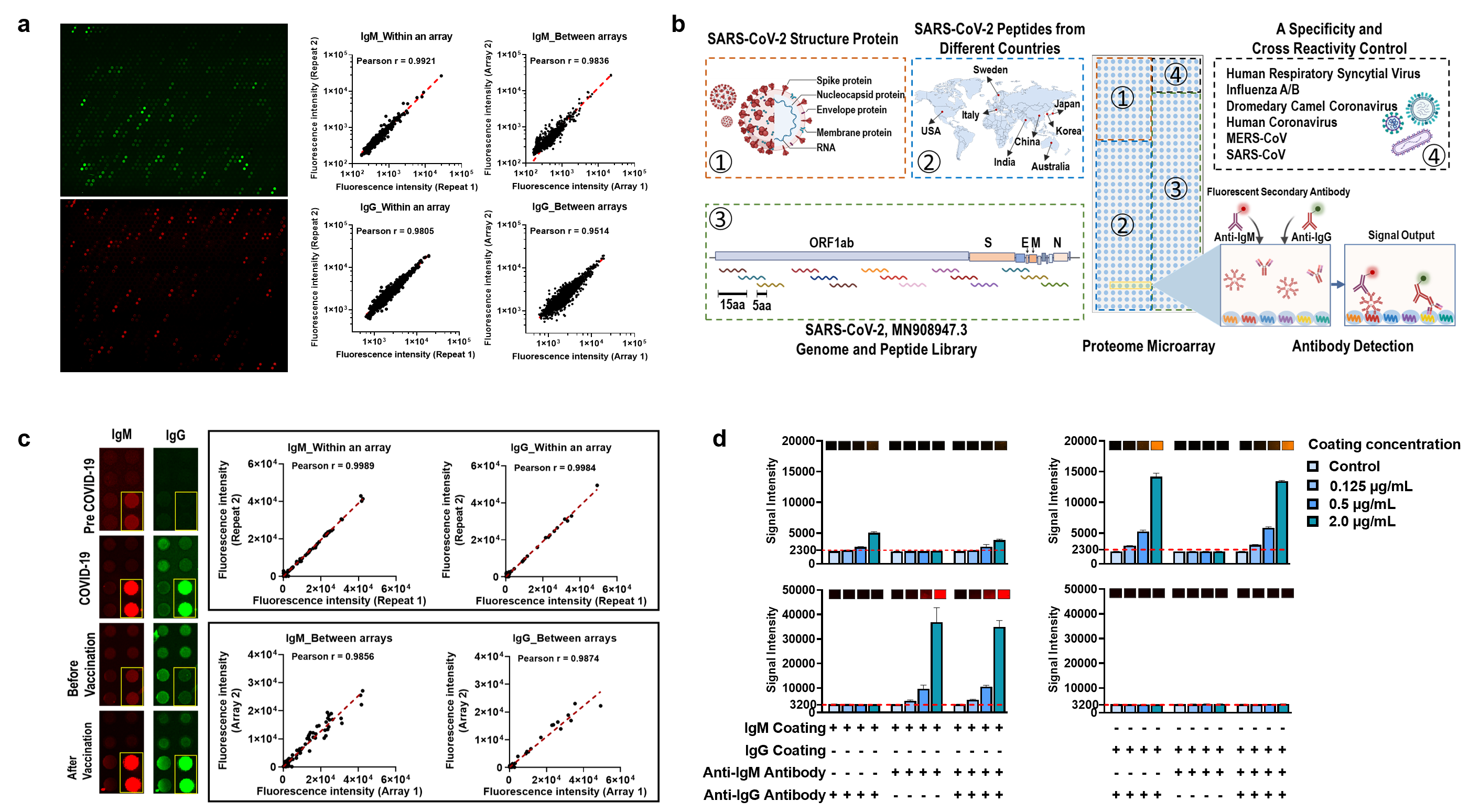


**Figure S2. Composition and performance of the one one-strain SARS-CoV-2 proteome microarray and the international SARS-CoV-2 microarray, related to Fig.1 and Fig.2.**

**a.** Reproducibility of antibody detection using the one-strain SARS-CoV-2 proteome microarray for non-human primates infected by SARS-CoV-2.

**b.** Schematic of the design, composition, and workflow of the international SARS-CoV-2 microarray.

**c.** Reproducibility of antibody detection using the international SARS-CoV-2 microarray for COVID-19 patients and vaccinated participants.

**d.** Cross reactivity of anti-IgG and anti-IgM secondary antibodies in employed in the international SARS-CoV-2 microarray.

Created with biorender.com.

**
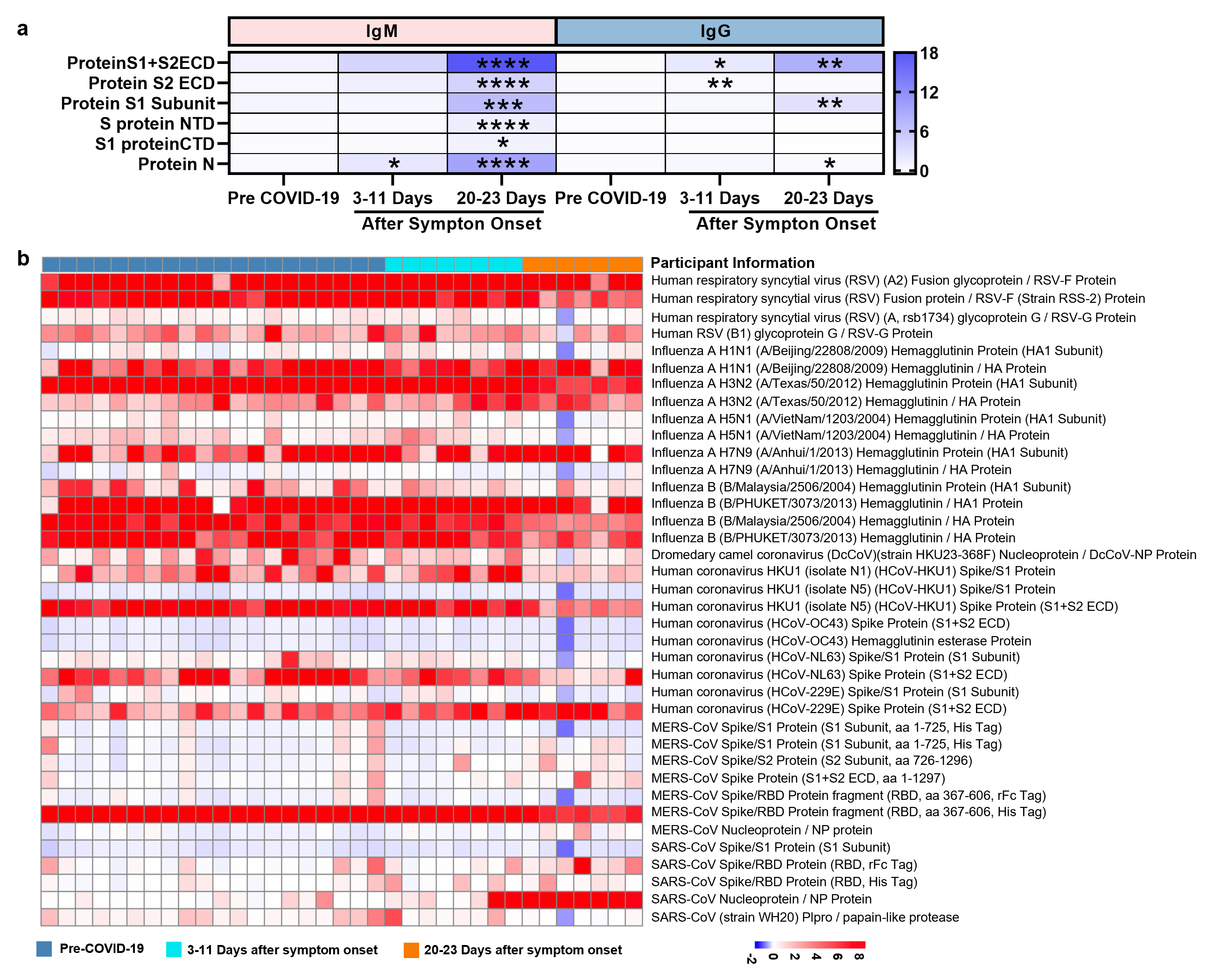
Figure S3. Heatmap of the mean IgM and/or IgG antibody signal detected for the indicated proteins and protein fragments of SARS-CoV-2 or other respiratory viruses using serum from the COVID-19 patient cohort, related to Fig.2.**

**a.** Heatmap of mean IgM and IgG signal detected for the indicated SARS-CoV-2 proteins and protein fragments.

**b.** Heatmap of the IgM signal detected for protein or protein fragments of the indicated respiratory viruses.


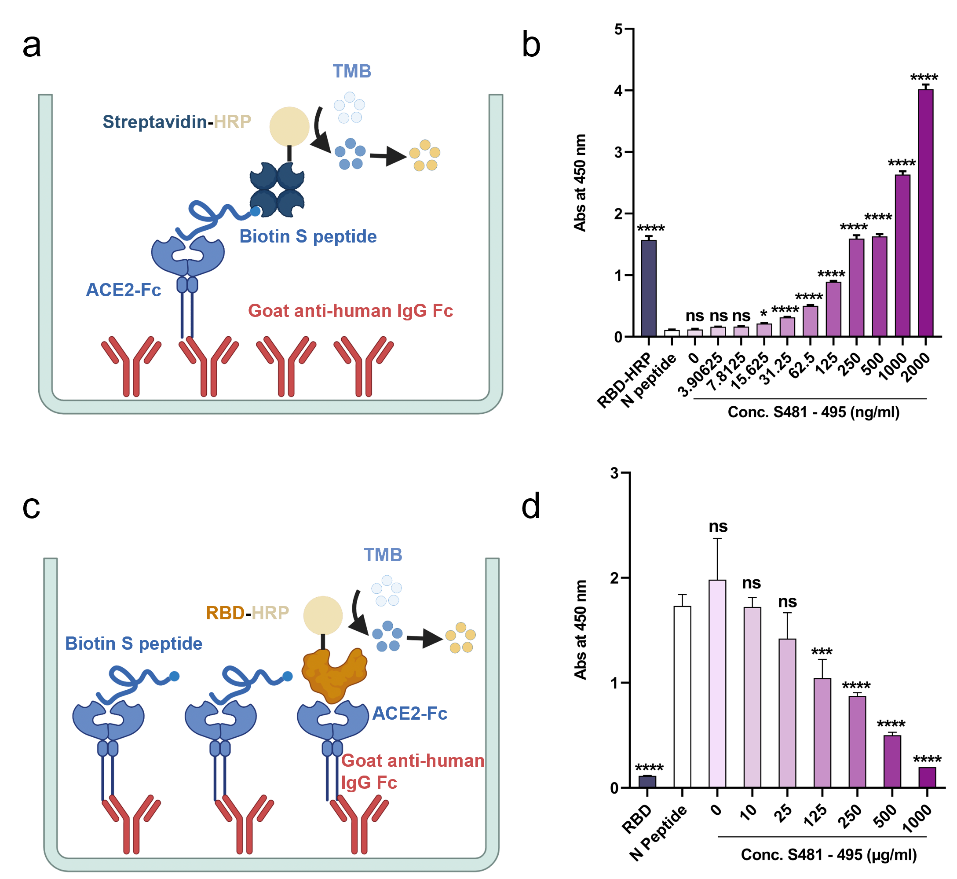


**Figure S4. An S protein LPE consistently detected by antibodies present in SARS-CoV-2-infected NHPs and patients (S481-495) binds to ACE2 and blocks its interaction with the S protein RBD.**

**a.** Schematic of an ELISA to measure S481-495 peptide binding to a recombinant ACE2 protein.

**b.** ELISA results for the binding of serial dilutions of the SARS-CoV-2 spike protein RBD, S481-485 peptide (0-2000 ng/mL), or N protein N161-175 peptide (500 ng/mL, negative control) to recombinant ACE2-Fc2 protein.

**c.** Schematic on a competitive ELISA to measure the ability of input N and S protein peptides to block the binding of the SARS-CoV-2 RBD with ACE2.

**d.** Competitive ELISA results for the signal detected when unlabeled SARS-CoV-2 RBD protein (20 µg/mL), S481-485 peptide (0-1000 µg/mL), or N protein N161-175 peptide (500 µg/mL, negative control) were used to block the interaction of HRP-labeled RDB with recombinant ACE2-Fc2 protein.

Graphs indicate Mean±SD values. P-values for differences with the N peptide group were determined by one-way ANOVA with Dunnett’s post-test (n=3/sample). ns: no significant, *p<0.05, **p<0.01, ***p<0.001, and **** p<0.0001. Created with biorender.com.

**Figure S5. Heatmap of the mean IgM and/or IgG antibody signal detected for the indicated proteins and protein fragments of SARS-CoV-2 or other respiratory viruses using serum from five individuals in vaccine cohort with longitudinal samples, related to Fig.3.a-b.**

**a, b.** Heatmap of mean IgG signal detected for a) the indicated SARS-CoV-2 proteins and protein fragments, or b) peptides corresponding to the indicated S protein sequence regions.

**c.** Heatmap of the IgM signal detected for protein or protein fragments of the indicated respiratory viruses.

| **Table S6. S protein epitope clusters detected in NHP, COVID-19 and vaccinated participants** | | | |
| --- | --- | --- | --- |
| Cluster | NHP | COVID-19 patients | Vaccinated participants |
|  | 121 | -- | 121 |
|  | -- | -- | 221 |
|  | 241 | 241 | -- |
|  | 301 | 301 | -- |
|  |  |  |  |
|  | 351 | -- | 351 |
|  | 431 | -- | 431 |
|  | 481 | 481 | -- |
| Cluster1 | 541 | -- | 541 |
|  | 551 | 551 | 551 |
|  | 561 | -- | 561 |
|  | -- | 571 | 571 |
| Cluster2 | 621 | 621 | 621 |
|  | 631 | -- | 631 |
| Cluster3 | 661 | 661 | -- |
|  | 691 | 691 | -- |
| Cluster4 | -- | 761 | -- |
|  | 781 | 781 | 781 |
|  | 791 | 791 | -- |
|  | 801 | 801 | -- |
|  | -- | 811 | 811 |
|  | -- | 921 | -- |
|  | -- | -- | 1001 |
|  | -- | 1101 | -- |
| Cluster5 | -- | -- | 1121 |
|  | -- | -- | 1161 |
|  | -- | -- | 1181 |
|  | -- | 1251 | -- |
| Numbers indicate S protein starting positions of detected peptides | | | |

| **Table S7. IgM S peptide among the COVID-19 patients, vaccinated and VPI participants** | | | |
| --- | --- | --- | --- |
| Domain | COVID-19 patients | Vaccinated participants | VPI participants |
| S1 NTD | -- | S121-135 | -- |
|  | -- | -- | S161-175 |
|  | -- | S221-235 | -- |
|  | S241-255 | -- | -- |
|  | -- | -- | S291-305 |
|  | S301-315 | -- | -- |
| RBD | -- | -- | S321-335 |
|  | -- | S351-365 | -- |
|  | -- | S431-445 | S431-445 |
|  | S481-495 | -- | -- |
|  | -- | -- | S501-515 |
|  | -- | -- | S511-525 |
|  | -- | S541-555 | -- |
| S1 CTD | S551-565 | S551-565 | S551-565 |
|  | -- | S561-575 | S561-575 |
|  | S571-586 | S571-588 | S571-587 |
|  | S621-635 | S621-635 | S621-635 |
|  | -- | S631-645 | S631-645 |
|  | -- | -- | S641-655 |
|  | S661-675 | S661-675 | S661-675 |
| S2 NTD | S691-705 | -- | S691-705 |
|  | S761-775 | -- | -- |
|  | S781-795 | S781-795 | S781-795 |
|  | S791-805 | -- | S791-805 |
|  | S801-815 | -- | -- |
|  | S811-825 | S811-825 | -- |
| HR1 | S921-935 | -- | -- |
| CH | -- | S1001-1015 | S1001-1015 |
|  | -- | -- | S1011-1025 |
| S2 Internal | S1101-1115 | -- | -- |
|  | -- | S1121-1135 | -- |
|  | -- | -- | S1141-1155 |
| HR2 | -- | S1161-1175 | S1161-1175 |
|  | -- | -- | S1171-1185 |
|  | -- | S1181-1195 | -- |
| CTD | S1251-1265 | -- | S1251-1265 |
|  | -- | -- | S1261-1273 |
| Numbers indicate S protein starting positions of detected peptides. Labels indicate the N-terminal, ribosome binding, and C-terminal domains (NTD, RBD, CTD) of the S1 or S2 region and the central helix (CH), heptad repeat 1 and 2 (HR1 and HR2) of the S2 region. VPI: Vaccination post infection. | | | |
