# Supplementary figures and images for: SARS-CoV-2 Epitopes following Infection and Vaccination Overlap Known Neutralizing Antibody Sites
Source: Research (Wash D C). 2022 Jul 9;2022:9769803. doi: 10.34133/2022/9769803 (PMC9297724; doi:10.34133/2022/9769803)

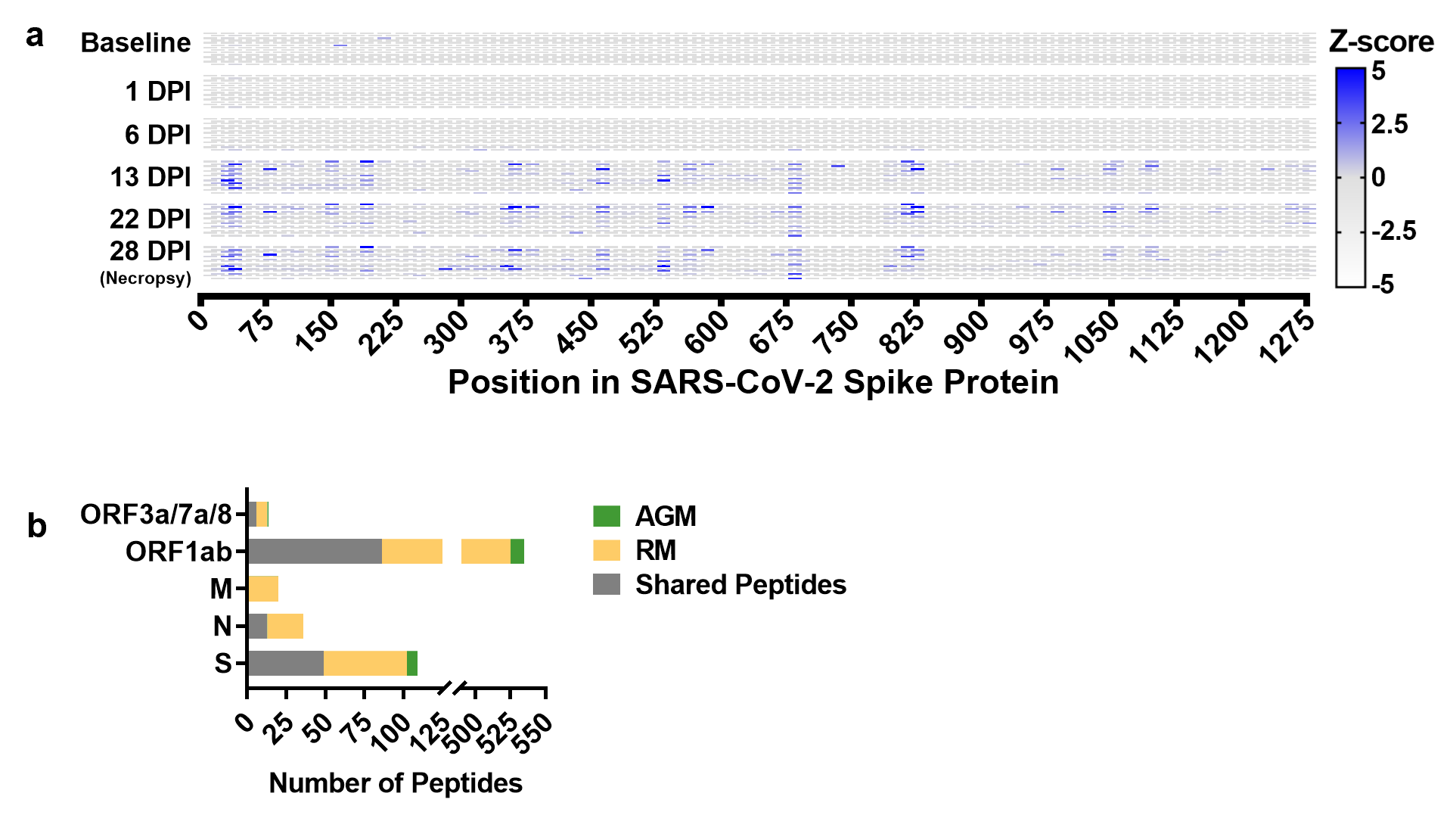

Supplement: Supplementary Materials — Figures S1: the IgM signal intensity of spike peptide positions with individual monkeys and relative abundance and overlap among IgM linear epitopes detected in the SARS-CoV-2 NHP models with different species, related to Figure 1. Figures S2: composition and performance of the one one-strain SARS-CoV-2 proteome microarray and the international SARS-CoV-2 microarray, related to Figures 1 and 2. Figures S3: heatmap of the mean IgM and/or IgG antibody signal detected for the indicated proteins and protein fragments of SARS-CoV-2 or other respiratory viruses using serum from the COVID-19 patient cohort, related to Figure 2. Figure S4: an S protein LPE consistently detected by antibodies present in SARS-CoV-2-infected NHPs and patients (S481-495) binds to ACE2 and blocks its interaction with the S protein RBD. Figures S5: heatmap of the mean IgM and/or IgG antibody signal detected for the indicated proteins and protein fragments of SARS-CoV-2 or other respiratory viruses using serum from five individuals in vaccine cohort with longitudinal samples, related to Figures 3(a) and 3(b). Table S6: S protein epitope clusters detected in NHP, COVID-19, and vaccinated participants. Table S7: IgM S peptide among the COVID-19 patients, vaccinated and VPI participants. Other supplementary materials for this manuscript include the following: dataset S1. Table S1: composition of the one-strain SARS-CoV-2 proteome microarray and the international SARS-CoV-2 proteome microarray, related to Figures 1–3. Dataset S2. Table S2: identified peptides specific to anti-IgM and anti-IgG from nonhuman primates infected with SARS-CoV-2, related to Figure 1. Dataset S3. Table S3: proteins and peptides detected by COVID-19 patient IgM and IgG, related to Figure 3. Dataset S4. Table S4: IgM binding to overlapping SARS-CoV-2 peptides in COVID-19 patients, related to Figure 2. Dataset S5. Table S5: IgM- and IgG-specific targets recognized by longitudinal vaccine participants and crosssection vaccinated [file 9769803.f1.zip › FigureS1.tif]

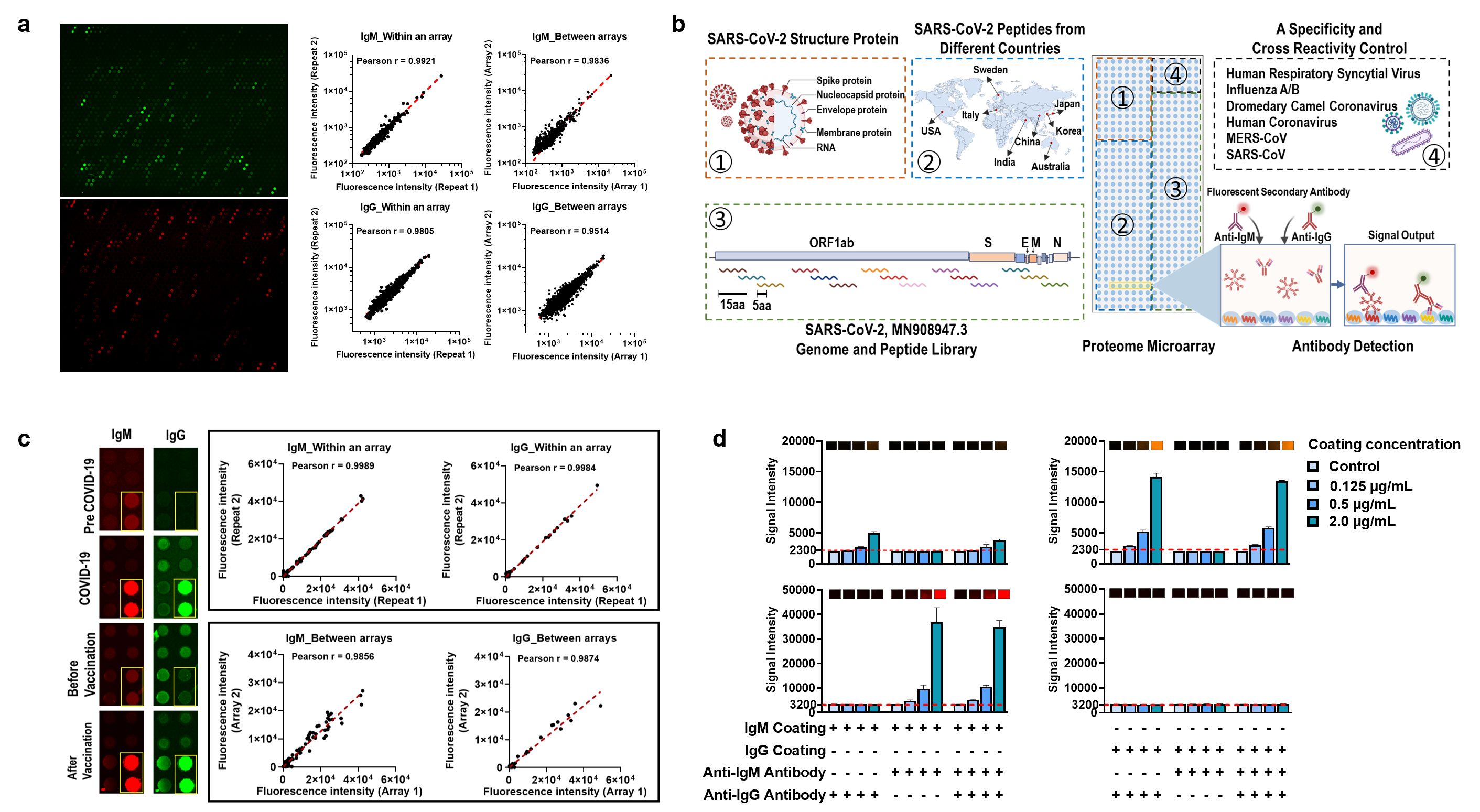

Supplement: Supplementary Materials — Figures S1: the IgM signal intensity of spike peptide positions with individual monkeys and relative abundance and overlap among IgM linear epitopes detected in the SARS-CoV-2 NHP models with different species, related to Figure 1. Figures S2: composition and performance of the one one-strain SARS-CoV-2 proteome microarray and the international SARS-CoV-2 microarray, related to Figures 1 and 2. Figures S3: heatmap of the mean IgM and/or IgG antibody signal detected for the indicated proteins and protein fragments of SARS-CoV-2 or other respiratory viruses using serum from the COVID-19 patient cohort, related to Figure 2. Figure S4: an S protein LPE consistently detected by antibodies present in SARS-CoV-2-infected NHPs and patients (S481-495) binds to ACE2 and blocks its interaction with the S protein RBD. Figures S5: heatmap of the mean IgM and/or IgG antibody signal detected for the indicated proteins and protein fragments of SARS-CoV-2 or other respiratory viruses using serum from five individuals in vaccine cohort with longitudinal samples, related to Figures 3(a) and 3(b). Table S6: S protein epitope clusters detected in NHP, COVID-19, and vaccinated participants. Table S7: IgM S peptide among the COVID-19 patients, vaccinated and VPI participants. Other supplementary materials for this manuscript include the following: dataset S1. Table S1: composition of the one-strain SARS-CoV-2 proteome microarray and the international SARS-CoV-2 proteome microarray, related to Figures 1–3. Dataset S2. Table S2: identified peptides specific to anti-IgM and anti-IgG from nonhuman primates infected with SARS-CoV-2, related to Figure 1. Dataset S3. Table S3: proteins and peptides detected by COVID-19 patient IgM and IgG, related to Figure 3. Dataset S4. Table S4: IgM binding to overlapping SARS-CoV-2 peptides in COVID-19 patients, related to Figure 2. Dataset S5. Table S5: IgM- and IgG-specific targets recognized by longitudinal vaccine participants and crosssection vaccinated [file 9769803.f1.zip › FigureS2.tif]

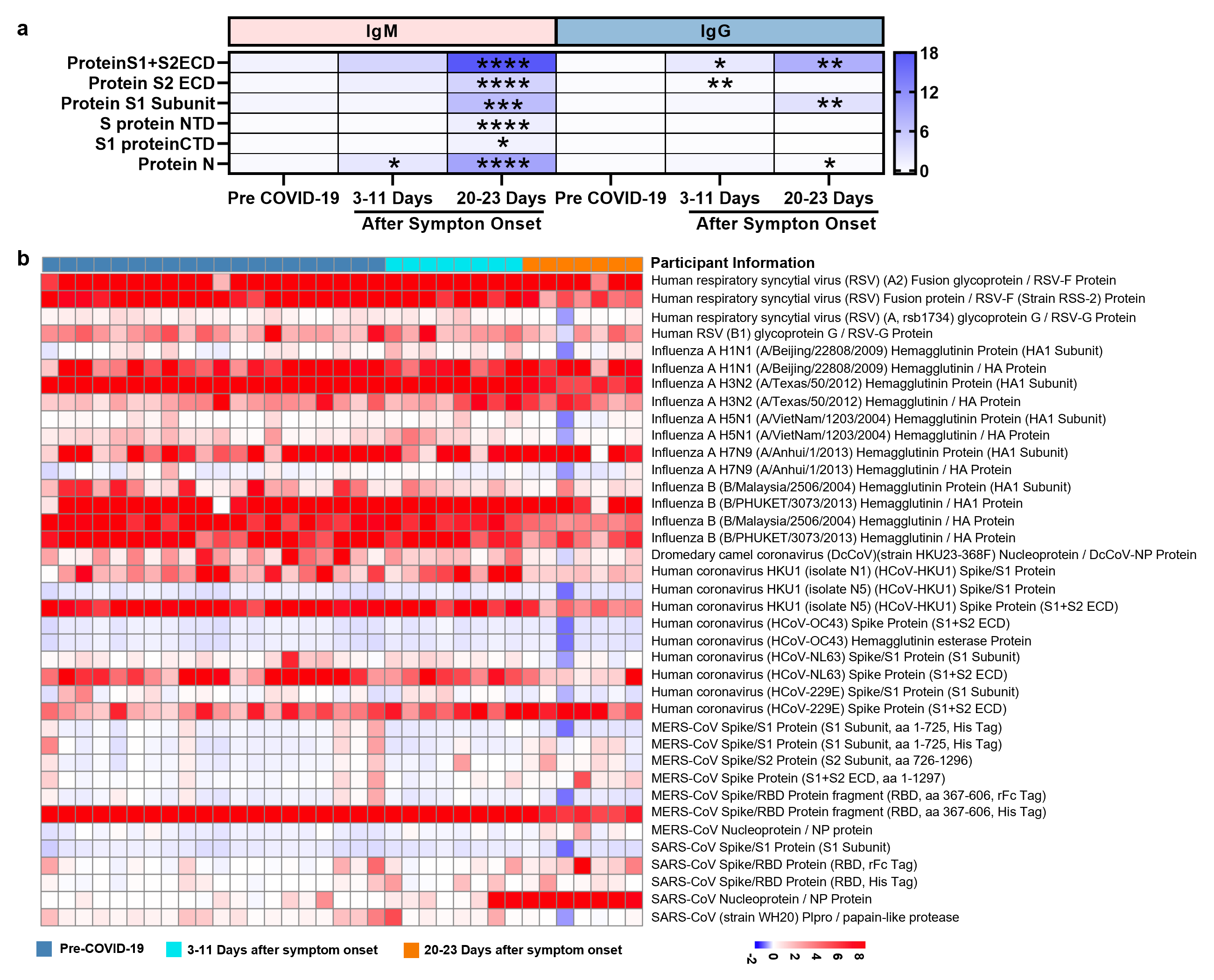

Supplement: Supplementary Materials — Figures S1: the IgM signal intensity of spike peptide positions with individual monkeys and relative abundance and overlap among IgM linear epitopes detected in the SARS-CoV-2 NHP models with different species, related to Figure 1. Figures S2: composition and performance of the one one-strain SARS-CoV-2 proteome microarray and the international SARS-CoV-2 microarray, related to Figures 1 and 2. Figures S3: heatmap of the mean IgM and/or IgG antibody signal detected for the indicated proteins and protein fragments of SARS-CoV-2 or other respiratory viruses using serum from the COVID-19 patient cohort, related to Figure 2. Figure S4: an S protein LPE consistently detected by antibodies present in SARS-CoV-2-infected NHPs and patients (S481-495) binds to ACE2 and blocks its interaction with the S protein RBD. Figures S5: heatmap of the mean IgM and/or IgG antibody signal detected for the indicated proteins and protein fragments of SARS-CoV-2 or other respiratory viruses using serum from five individuals in vaccine cohort with longitudinal samples, related to Figures 3(a) and 3(b). Table S6: S protein epitope clusters detected in NHP, COVID-19, and vaccinated participants. Table S7: IgM S peptide among the COVID-19 patients, vaccinated and VPI participants. Other supplementary materials for this manuscript include the following: dataset S1. Table S1: composition of the one-strain SARS-CoV-2 proteome microarray and the international SARS-CoV-2 proteome microarray, related to Figures 1–3. Dataset S2. Table S2: identified peptides specific to anti-IgM and anti-IgG from nonhuman primates infected with SARS-CoV-2, related to Figure 1. Dataset S3. Table S3: proteins and peptides detected by COVID-19 patient IgM and IgG, related to Figure 3. Dataset S4. Table S4: IgM binding to overlapping SARS-CoV-2 peptides in COVID-19 patients, related to Figure 2. Dataset S5. Table S5: IgM- and IgG-specific targets recognized by longitudinal vaccine participants and crosssection vaccinated [file 9769803.f1.zip › FigureS3.tif]

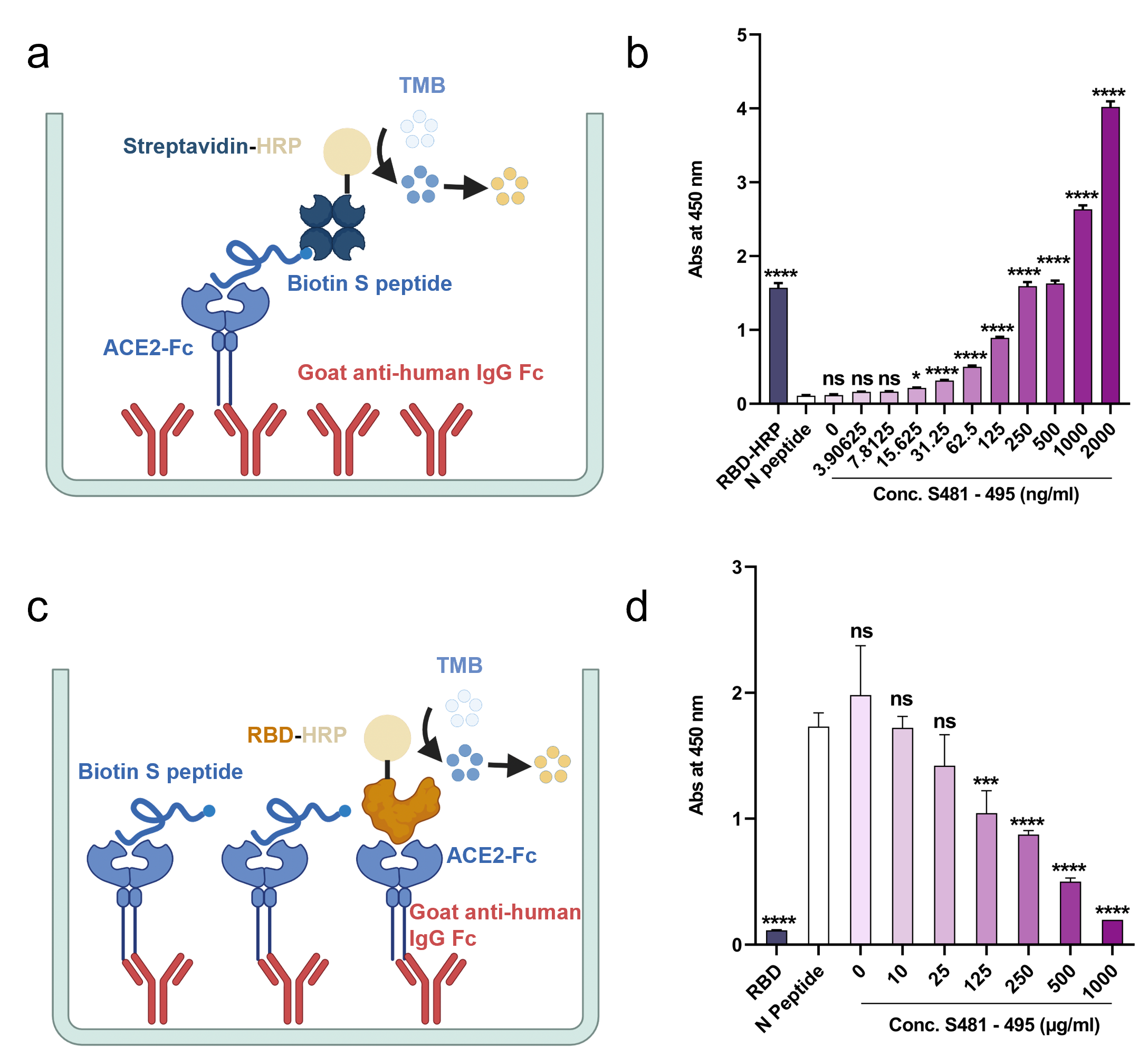

Supplement: Supplementary Materials — Figures S1: the IgM signal intensity of spike peptide positions with individual monkeys and relative abundance and overlap among IgM linear epitopes detected in the SARS-CoV-2 NHP models with different species, related to Figure 1. Figures S2: composition and performance of the one one-strain SARS-CoV-2 proteome microarray and the international SARS-CoV-2 microarray, related to Figures 1 and 2. Figures S3: heatmap of the mean IgM and/or IgG antibody signal detected for the indicated proteins and protein fragments of SARS-CoV-2 or other respiratory viruses using serum from the COVID-19 patient cohort, related to Figure 2. Figure S4: an S protein LPE consistently detected by antibodies present in SARS-CoV-2-infected NHPs and patients (S481-495) binds to ACE2 and blocks its interaction with the S protein RBD. Figures S5: heatmap of the mean IgM and/or IgG antibody signal detected for the indicated proteins and protein fragments of SARS-CoV-2 or other respiratory viruses using serum from five individuals in vaccine cohort with longitudinal samples, related to Figures 3(a) and 3(b). Table S6: S protein epitope clusters detected in NHP, COVID-19, and vaccinated participants. Table S7: IgM S peptide among the COVID-19 patients, vaccinated and VPI participants. Other supplementary materials for this manuscript include the following: dataset S1. Table S1: composition of the one-strain SARS-CoV-2 proteome microarray and the international SARS-CoV-2 proteome microarray, related to Figures 1–3. Dataset S2. Table S2: identified peptides specific to anti-IgM and anti-IgG from nonhuman primates infected with SARS-CoV-2, related to Figure 1. Dataset S3. Table S3: proteins and peptides detected by COVID-19 patient IgM and IgG, related to Figure 3. Dataset S4. Table S4: IgM binding to overlapping SARS-CoV-2 peptides in COVID-19 patients, related to Figure 2. Dataset S5. Table S5: IgM- and IgG-specific targets recognized by longitudinal vaccine participants and crosssection vaccinated [file 9769803.f1.zip › FigureS4.tif]

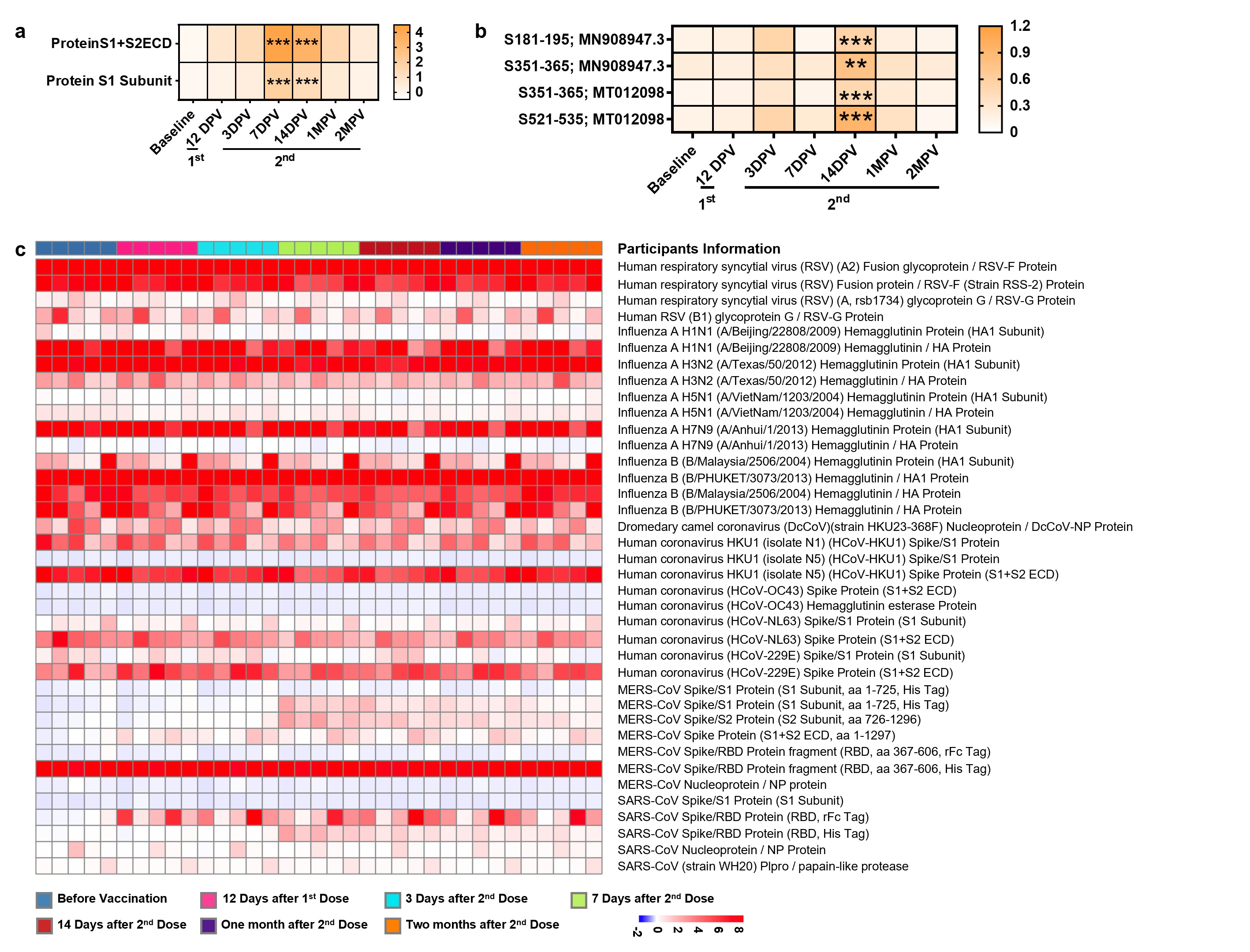

Supplement: Supplementary Materials — Figures S1: the IgM signal intensity of spike peptide positions with individual monkeys and relative abundance and overlap among IgM linear epitopes detected in the SARS-CoV-2 NHP models with different species, related to Figure 1. Figures S2: composition and performance of the one one-strain SARS-CoV-2 proteome microarray and the international SARS-CoV-2 microarray, related to Figures 1 and 2. Figures S3: heatmap of the mean IgM and/or IgG antibody signal detected for the indicated proteins and protein fragments of SARS-CoV-2 or other respiratory viruses using serum from the COVID-19 patient cohort, related to Figure 2. Figure S4: an S protein LPE consistently detected by antibodies present in SARS-CoV-2-infected NHPs and patients (S481-495) binds to ACE2 and blocks its interaction with the S protein RBD. Figures S5: heatmap of the mean IgM and/or IgG antibody signal detected for the indicated proteins and protein fragments of SARS-CoV-2 or other respiratory viruses using serum from five individuals in vaccine cohort with longitudinal samples, related to Figures 3(a) and 3(b). Table S6: S protein epitope clusters detected in NHP, COVID-19, and vaccinated participants. Table S7: IgM S peptide among the COVID-19 patients, vaccinated and VPI participants. Other supplementary materials for this manuscript include the following: dataset S1. Table S1: composition of the one-strain SARS-CoV-2 proteome microarray and the international SARS-CoV-2 proteome microarray, related to Figures 1–3. Dataset S2. Table S2: identified peptides specific to anti-IgM and anti-IgG from nonhuman primates infected with SARS-CoV-2, related to Figure 1. Dataset S3. Table S3: proteins and peptides detected by COVID-19 patient IgM and IgG, related to Figure 3. Dataset S4. Table S4: IgM binding to overlapping SARS-CoV-2 peptides in COVID-19 patients, related to Figure 2. Dataset S5. Table S5: IgM- and IgG-specific targets recognized by longitudinal vaccine participants and crosssection vaccinated [file 9769803.f1.zip › FigureS5.tif]
